# The Underestimation of Global Microbial Diversity

**DOI:** 10.1128/mBio.01298-16

**Published:** 2016-09-27

**Authors:** Jay T. Lennon, Kenneth J. Locey

**Affiliations:** Department of Biology, Indiana University, Bloomington, Indiana, USA

## LETTER

In a recent commentary, Amann and Rosselló-Móra summarized how the census of *Bacteria* and *Archaea* has changed over time (1). For decades, the number of recognized microbial taxa was underestimated owing to limitations associated with culture-based methods and the rules of nomenclature. The authors describe a “quantum leap” in the estimates of global microbial diversity following advances in high-throughput sequencing technology. Despite this, Amann and Rosselló-Móra project that a complete census of microbial diversity will be reached within a few years, culminating in the lower millions of taxa (also, see reference 2). While perhaps attractively optimistic to some, this presumption is misleading for the following reasons.

First, some data sets reveal that global microbial diversity has already surpassed the lower-million estimate suggested by Amann and Rosselló-Móra (1). For example, in August of 2014, there were 5.6 million operational taxonomic units (OTUs) based on the open-reference database of short-read 16S rRNA gene sequences from the Earth Microbiome Project. This number has doubled to over 11 million OTUs in less than 2 years (3). The majority of these taxa have only been detected once or twice, suggesting that earth’s microbiome remains greatly undersampled.

Second, one must be cautious when making estimates of diversity based on extrapolations from rarefaction and accumulation curves. For example, the majority of full-length 16S rRNA gene sequences deposited over the last decade come from a small number of studies in a limited range of habitats. In most of those years, there were less than 11 submissions, while in some years there were as few as 2 submissions (4). Such efforts are insufficient for inferring microbial diversity in environmental, managed, and host-associated ecosystems across the planet (4). We argue that the microbial census will expand if other ecosystems are sampled with more effort. For example, just last year, 35 new candidate phyla were recovered in groundwater from a single aquifer well (5).

Finally, the recent census states that ongoing efforts are doing a good job of capturing the most abundant organisms but struggle with the sampling of rarer organisms (4). However, Amann and Rosselló-Móra cast doubt on the contribution of rare taxa to global microbial biodiversity by stating “The tail observed in rank abundance curves could be not so long, after all” (1). In contrast to this view, our recent findings suggest that the rare biosphere is likely a large reservoir of species diversity in microbial systems. Using a large compilation of macrobial (plant and animal) and microbial data, we demonstrated that rarity increases with the number of individuals (*N*) in a system (6). This finding is consistent with theoretical expectations that low-abundance taxa are more prevalent in systems with a greater *N* (7). On a planet with an estimated 10^30^ individuals, we predict that most bacterial and archaeal species are rare but essential for generating an accurate microbial census.
